# CD, UV, and In Silico Insights on the Effect of 1,3-Bis(1′-uracilyl)-2-propanone on Serum Albumin Structure

**DOI:** 10.3390/biom12081071

**Published:** 2022-08-03

**Authors:** Francesca Greco, Andrea Patrizia Falanga, Monica Terracciano, Carlotta D’Ambrosio, Gennaro Piccialli, Giorgia Oliviero, Giovanni Nicola Roviello, Nicola Borbone

**Affiliations:** 1 Department of Pharmacy, University of Naples Federico II, Via Domenico Montesano 49, 80131 Naples, Italy; francesca.greco@unina.it (F.G.); andreapatrizia.falanga@unina.it (A.P.F.); monica.terracciano@unina.it (M.T.); carlottada1995@gmail.com (C.D.); picciall@unina.it (G.P.); nicola.borbone@unina.it (N.B.); 2 Institute of Applied Sciences and Intelligent Systems “Eduardo Caianiello”, Italian National Council of Research (ISASI-CNR), Via Pietro Castellino 111, 80131 Naples, Italy; 3 ISBE-IT, University of Naples Federico II, Corso Umberto I, 80138 Naples, Italy; golivier@unina.it; 4 Department of Molecular Medicine and Medical Biotechnologies, University of Naples Federico II, Via Sergio Pansini 5, 80131 Naples, Italy; 5 Institute of Biostructures and Bioimaging, Italian National Council for Research (IBB-CNR), Area di Ricerca Site and Headquarters, Via Pietro Castellino 111, 80131 Naples, Italy

**Keywords:** serum albumin, 1,3-diaryl-2-propanone, 1,3-bis(1′-uracilyl)-2-propanone, circular dichroism, molecular docking, protein–ligand interactions

## Abstract

1,3-diaryl-2-propanone derivatives are synthetic compounds used as building blocks for the realization not only of antimicrobial drugs but also of new nanomaterials thanks to their ability to self-assemble in solution and interact with nucleopeptides. However, their ability to interact with proteins is a scarcely investigated theme considering the therapeutic importance that 1,3-diaryl-2-propanones could have in the modulation of protein-driven processes. Within this scope, we investigated the protein binding ability of 1,3-bis(1′-uracilyl)-2-propanone, which was previously synthesized in our laboratory utilizing a Dakin–West reaction and herein indicated as U2O, using bovine serum albumin (BSA) as the model protein. Through circular dichroism (CD) and UV spectroscopy, we demonstrated that the compound, but not the similar thymine derivative T2O, was able to alter the secondary structure of the serum albumin leading to significant consequences in terms of BSA structure with respect to the unbound protein (Δ_β-turn_ + Δ_β-sheet_ = +23.6%, Δ_α_ = −16.7%) as revealed in our CD binding studies. Moreover, molecular docking studies suggested that U2O is preferentially housed in the domain IIIB of the protein, and its affinity for the albumin is higher than that of the reference ligand HA 14−1 (HDOCK score (top 1–3 poses): −157.11 ± 1.38 (U2O); −129.80 ± 6.92 (HA 14−1); binding energy: −7.6 kcal/mol (U2O); −5.9 kcal/mol (HA 14−1)) and T2O (HDOCK score (top 1–3 poses): −149.93 ± 2.35; binding energy: −7.0 kcal/mol). Overall, the above findings suggest the ability of 1,3-bis(1′-uracilyl)-2-propanone to bind serum albumins and the observed reduction of the α-helix structure with the concomitant increase in the β-structure are consistent with a partial protein destabilization due to the interaction with U2O.

## 1. Introduction

**Figure 1:**
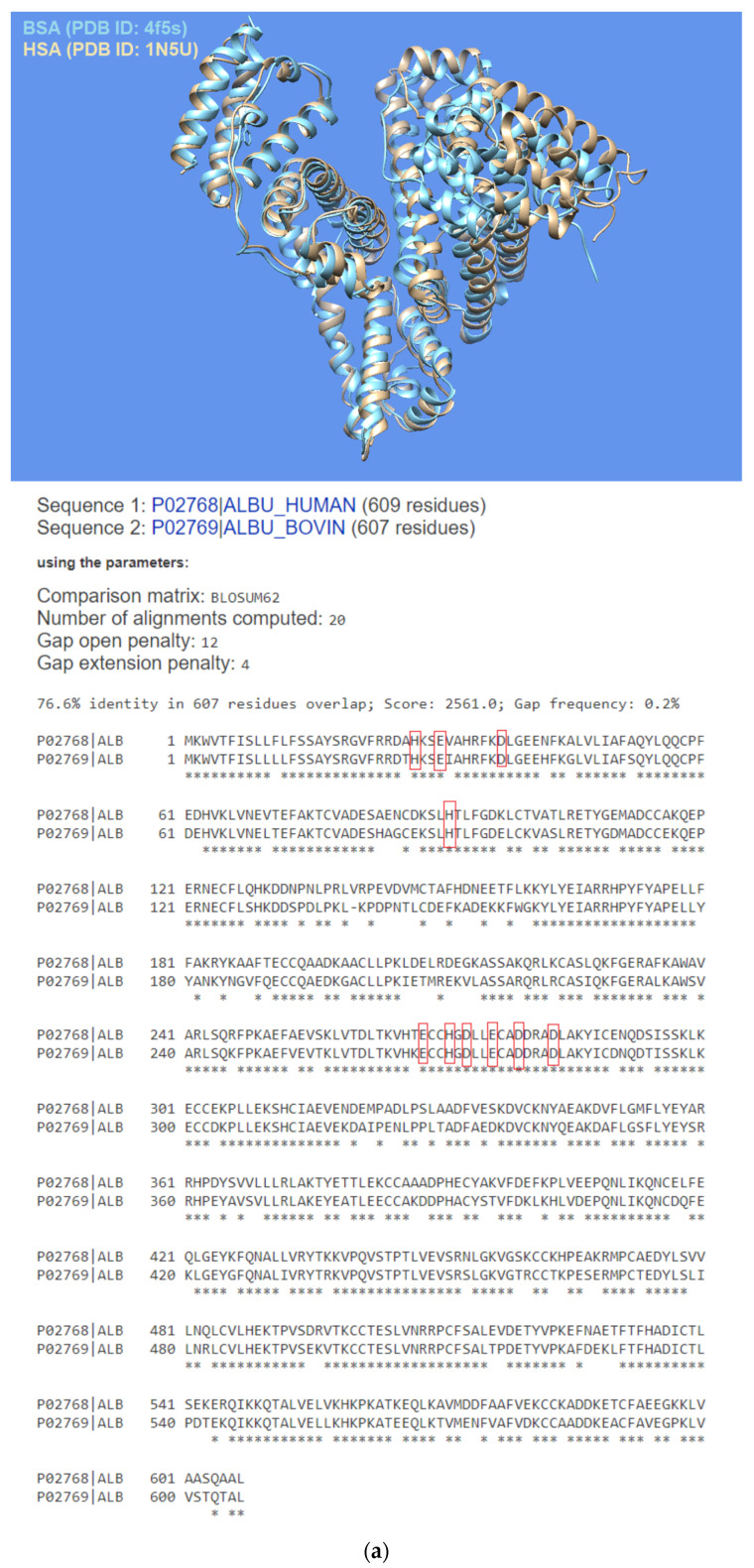

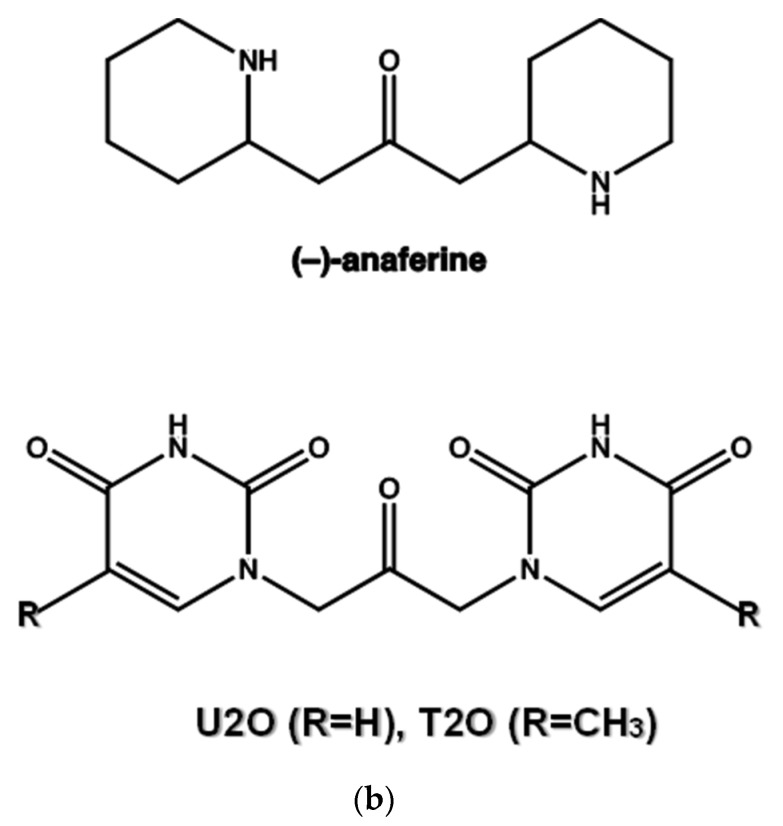
(**a**) Superimposed structures of HSA (PDB ID: 1n5u, light brown) and BSA (PDB ID: 4f5s, cyan) as visualized by UCSF Chimera software (Resource for Biocomputing, Visualization, and Informatics at the University of California, San Francisco, CA, USA, v.1.14) [49] and protein sequence alignment for HSA (P02768) and BSA (P02769) as obtained by Expasy software (Swiss Institute of Bioinformatics, Geneva, Switzerland, SIM-Alignment Tool for protein sequences) (https://web.expasy.org/sim/, accessed on 14 July 2022) with conserved binding site residues evidenced in red (https://www.uniprot.org/uniprotkb/P02768/entry and https://www.uniprot.org/uniprotkb/P02769/entry, accessed on 14 July 2022). (**b**) Chemical structures of the compounds studied as the protein ligands in the current work compared to (–)-anaferine.

Among the several families of proteins, albumins [1,2,3] are characterized by high peptide sequence homology, with human and bovine albumins sharing more than 75% identity [4]. Abundantly present in the circulatory system, albumins help maintain the osmotic blood pressure between the tissues and blood vessels [5]. Albumins, particularly bovine serum albumin (BSA), are often employed as protein models [6,7,8], and their interaction with most diverse ligands has been investigated for various applications in biomedicine and industrial areas [5,9,10,11,12,13,14,15]. The crystal structures of human serum albumin (HSA) and BSA, which share ~80% identity [16] and ~90% homology similarity [17] in the amino acid sequence, are shown superimposed in Figure 1a together with the sequence alignments. The albumin structure shows three domains with two main high-affinity binding sites alongside several others to which ligands bind with lower affinity [18].

In their primary physiological role, albumins transport many classes of molecules, such as metal ions, steroids, fatty acids, and amino acids, from the bloodstream to their specific target organs [4,19]. Remarkably, this function and the albumin’s ability to bind molecules with high affinity led to the pharmaceutical application of albumins as drug-carrying systems [20,21,22,23].

Heterocyclic compounds are an important class of molecules endowed with interesting biological functions and therapeutical potential, which are structurally related to different natural compounds [24,25,26]. Among others, benzofurans are potential anti-inflammatory [27], antibiotic [28,29,30], anticancer [31,32,33], neuroprotective and analgesic [34], and antiparasitic [35] compounds. The ability to bind nucleic acids [36] and inhibit specific serine/threonine kinases implied in cancer development (thus affecting the cancer cell cycle) were proposed as some of the most probable anticancer mechanisms for this class of molecules [32,37].

1,3-diaryl-2-propanone derivatives [38,39] are synthetic compounds used as building blocks for the realization not only of antimicrobial drugs [40] but also of new nanomaterials thanks to their ability to self-assemble in solution and interact with nucleopeptides [39,41]. Structurally, they are heteroaromatic analogs of (−)-anaferine, an alkaloid with sedative-hypnotic and anticancer properties extracted from Asiatic solanaceous plants (Figure 1b) [42]. Structural analogies can also be seen with certain 2-propanone derivatives 1,3-disubstituted with six-term rings (phenyl and piperazine rings) endowed with antimuscarinic properties [43]. In addition to the vast number of biomedical applications of 1,3-diaryl-2-propanones, our compound can be seen as an analog of diaryl urea derivatives that constitute an important family of anticancer drugs thanks to their ability to interact with specific proteins involved in the disease [44]. However, differently from diaryl ureas, the ability of 1,3-diaryl-2-propanone derivatives to interact with proteins remains a theme still scarcely explored and worthy of further research for the therapeutic importance that 1,3-diaryl-2-propanones could have in the modulation of protein-driven processes. With this in mind, we decided to investigate the protein binding ability of 1,3-bis(1′-uracilyl)-2-propanone, which was previously synthesized in our laboratory [41] utilizing the Dakin–West reaction [45,46,47,48] and herein indicated as U2O [41] (Figure 1b), using BSA as the model protein. Moreover, a comparison with T2O [39], a closely related derivative carrying thymine moieties in place of uracil (Figure 1b), was performed by spectroscopy, as described in the sections below.

## 2. Materials and Methods

### 2.1. Synthesis of 1,3-Bis(1′-uracilyl)-2-propanone (U2O) and 1,3-Bis(1′-thyminyl)-2-propanone (T2O)

U2O and T2O were obtained with high purity (≥95%) as ascertained by HPLC analysis following a synthetic procedure described in the literature based on the Dakin–West reaction [39,41]. All the intermediates used in the synthesis were from Acros Organics (Thermo Scientific Chemicals, Waltham, MA, USA) and Sigma-Aldrich (Merck KGaA, Darmstadt, Germany) and used without further purification steps.

### 2.2. CD and UV Binding Studies

Circular dichroism (CD) spectra were recorded on a Jasco J-715 spectropolarimeter equipped with a Jasco Peltier PTC-423S/15 temperature controller (Jasco Europe S.r.l, Cremella, Italy) using a Hellma-238-QS tandem quartz cell (2 × 0.4375 cm from Hellma Italia S.r.l., Milano, Italy) according to the previous literature experiments [50,51,52,53,54,55,56,57,58,59] with a response time of 1 s, a scanning speed of 100 nm min^−1^, and a bandwidth of 2.0 nm in the 205–260 nm wavelength range. All the spectra were averaged over three scans. UV absorption spectra were collected simultaneously to CD on the same instrument [60] to minimize possible errors induced by separate measurements on different instruments [61]. CD (Δε) values (M^−1^ cm^−1^) were calculated according to the equation: Δε = θ/(32980 × C × l), with θ being the measured ellipticity (mdeg), C the concentration (M), and l the optical path length (cm). All experiments were performed at 10 °C in 10 mM of sodium phosphate buffer at pH 7.4. The concentration of BSA (Sigma-Aldrich) was 1.5 μM.

### 2.3. CD Spectra Deconvolution

For the deconvolution of the circular dichroism spectra, CD (mdeg) and wavelength (nm) data were given as input to the program CD3 (http://lucianoabriata.altervista.org/jsinscience/cd/cd3.html, accessed on 30 May 2022) [62,63]. Only data corresponding to positive coefficient values were selected for the protein structure analysis, choosing the “Fit 4 components” option. 

### 2.4. Molecular Docking and In Silico Protein–Ligand Interaction Analysis

Molecular docking (MD) simulations [7,64,65,66,67,68] were performed running the HDOCK server (http://hdock.phys.hust.edu.cn, accessed on 30 May 2022) [69,70], suitable for both macromolecule-to-macromolecule [69] and macromolecules-to-small molecules [71] rigid dockings, using default parameters. The PDB entry 4f5s [72] and the energy-minimized 3D structure models of U2O and T2O (obtained by MOLVIEW (http://molview.org, accessed on 30 May 2022), and saved as .pdb files) were uploaded into the HDOCK server as the target and ligands, respectively. Interestingly, the HDOCK server predicts the protein/ligand interaction through a hybrid algorithm of template-free docking (when PDB IDs/structures are furnished such as in our case) and template-based (when only the target sequence is furnished) and specifically uses the PDB ID: 1n5u present in its database as a template for the 3D structure of the albumin, which is endowed with the highest homological identity with respect to 4f5s [73]. Based on the structural similarity of HSA to BSA, docking of ligands into possible binding sites of BSA was previously performed using the crystal structure of HSA [73]. However, caution should be paid when considering binding sites such as Site 1 that are less conserved among the different albumins than Site 2, because this can lead to different positions of the ligands within the site and different binding efficiencies for the two albumins [74,75,76]. Since we furnished the PDB ID: 4f5s as an input target, our dockings were based on BSA and not HSA structure. Thanks to the iterative knowledge-based scoring function ITScore-PP, the HDOCK server ranked the top ten poses obtained after each docking run. The program’s energy score (HDOCK score) values predicted by ITScore-PP are dimensionless, with larger negative numbers indicating higher affinity interactions between the interacting ligand and the target macromolecule, which was previously reported to correlate well to experimental binding affinities with a correlation coefficient of R = 0.71 [77]. More details on the HDOCK docking server, including the procedures for the docking, can be found at http://hdock.phys.hust.edu.cn (accessed on 30 May 2022). We analyzed the top-ranked pose (Top-1) and the top three and ten ranked poses for the complexes predicted by HDOCK according to the energy scores provided by the program, as explained in the Results section. The protein–ligand interaction diagrams reported in this work were obtained by ProteinsPlus (https://proteins.plus/, accessed on 30 May 2022) [78]. The FireDock software (Tel Aviv University, Tel Aviv, Israel) [79] was then used for rescoring and refinement because of its ability to improve the flexibility and correct scoring errors typically experienced during the molecular docking calculations by fast rigid-body docking tools [79]. The top 100 results for the rigid BSA/U2O docking previously obtained by PatchDock, a docking program based on ligand–receptor geometric shape complementarity [80], were transferred to FireDock for refinement. The top-ranked FireDock solutions, according to the contribution of the atomic contact energy (ACE), were chosen for the study of the complexes. Binding energies (kcal/mol) were computed by AutoDock Vina using the 1-Click Mcule online platform (https://mcule.com (accessed on 14 July 2022, Mcule Inc., Palo Alto, CA, USA)) [81,82]. Details on the procedure with 1-Click Mcule including a tutorial are available at https://mcule.com/apps/1-click-docking/ (accessed on 14 July 2022). The x, y, z coordinates for the binding centers were those corresponding to Phe 550 (34.799, 13.656, 124.426) for the subdomain IIIB (U2O), Val 432 (11.840, 24.326, 120.374) for the subdomain IIIA (T2O), and Ser 191 (59.980, 20.440, 89.569) for the subdomain IIA (HA 14−1). 

### 2.5. Pharmacokinetic Properties

The SMILES (Simplified Molecular Input Line Entry System) codes of U2O (O=C(CN1C=CC(=O)NC1=O)CN2C=CC(=O)NC2=O) and T2O (Cc2cn(CC(=O)Cn1cc(C)c(=O)[nH]c1=O)c(=O)[nH]c2=O) were obtained with the MOLVIEW software (TU Delft, Deft, Netherlands, v2.4) and applied to calculate the logarithms of the partition coefficients (cLogP), blood-brain barrier (BBB) permeability, pan-assay interference compounds (PAINS) score, and druggability properties (Lipinski model) presented in this work by using the SwissADME web service (http://www.swissadme.ch/index.php, accessed on 30 May 2022).

## 3. Results and Discussion

**Figure 2:**
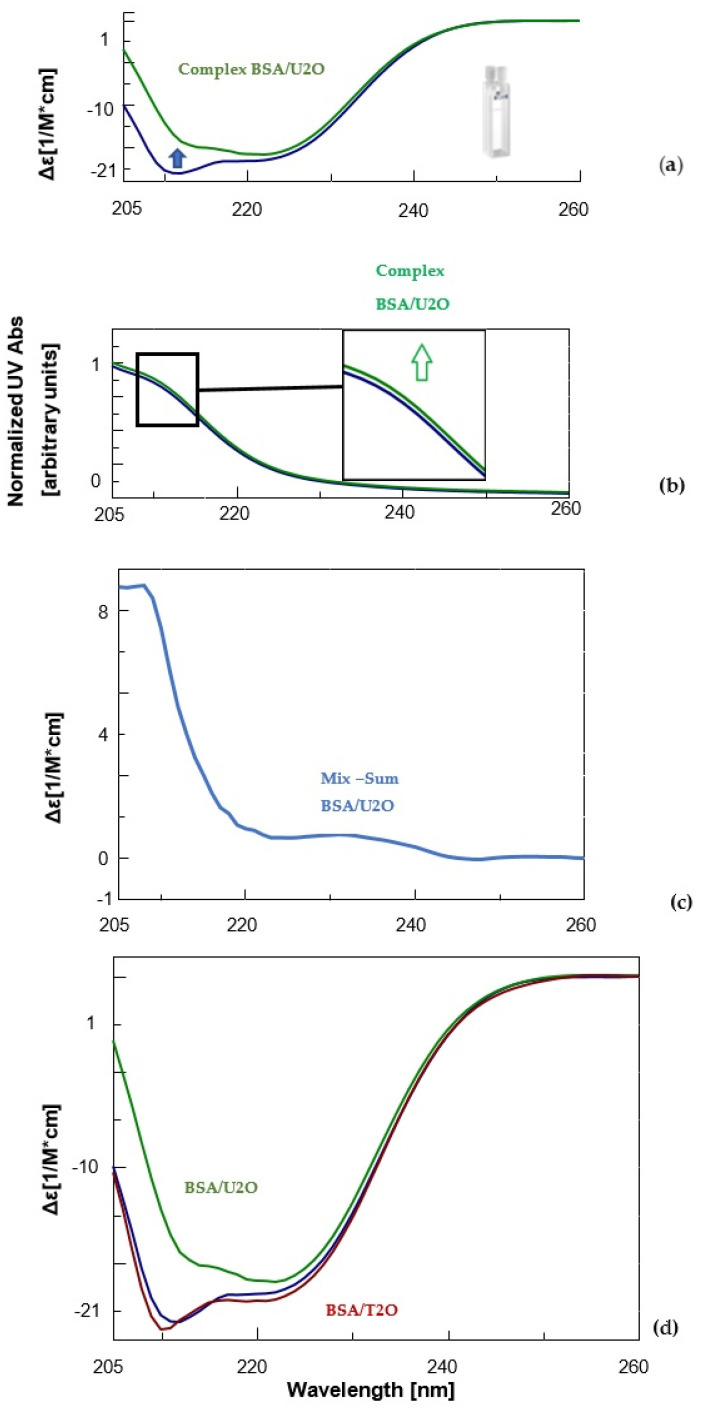
CD (**a**) and UV (**b**) spectra of BSA (1.5 μM, blue) and its complex with the 1,3-bis(1′-uracilyl)-2-propanone (6 nmol, green) in 10 mM sodium phosphate buffer (pH = 7.5) at 10 °C. Inset in Figure 2b, a zoomed-in view of UV bands between 210 and 215 nm. (**c**) Difference in CD spectrum of BSA in complex with U2O (CD_complex_ − CD_BSA_). (**d**) Comparison of the CD spectra of BSA (blue), its complex with U2O (green) and T2O (red) under the same experimental conditions previously indicated.

Experimental and computational (docking, ligand–protein analysis) studies were conducted to achieve insights into the molecular recognition of the model protein BSA by the U2O and T2O ligands. CD and UV experiments were performed in a two-chamber cell (Figure 2a) [83,84,85] in which we placed the protein and an excess of ligand separately. After mixing the solutions, the CD spectra of the two components (BSA and U[T]2O) were measured. The concentrations of both protein and ligand were halved after mixing, but the path length (2 × 0.4375 cm) increased by a factor of two. Any spectral difference observed after mixing the two component solutions was indicative of interaction [83,84,85].

### 3.1. Spectroscopic Binding Studies on BSA with U2O and T2O

Notoriously, the BSA structure is dominated by α-helix structures, which account for approximately 60% of its structure [86]. Moreover, about 20% of β-sheet + β-turn structures as well as ca. 20% random coil were experimentally observed for the native BSA in solution [87,88]. Accordingly, our far-UV CD experiments revealed for the unliganded serum albumin, mainly the characteristic features of the typical helical structure of proteins, i.e., two negative bands at about 208 and 222 nm (Figure 2a, blue line). After complexation with U2O, the CD band at 208 nm underwent bathochromic and hypochromic shifts (Figure 2a, green line). This evidence, together with the slight hyperchromic effect observed in the UV spectrum of the complex (Figure 2b, green line) relative to the curve of the unliganded BSA (blue line), and the non-null difference CD spectrum (Figure 2c), indicated that the BSA secondary structure underwent slight but clear modifications as a consequence of the interaction with U2O but not T2O (Figure 2d). In fact, when the CD binding experiment was performed with T2O, no substantial CD spectral difference was observed, suggesting that T2O was not able to provoke significant secondary structure perturbation in the albumin target. To achieve more quantitative information on the interaction of U2O with the protein, we performed a deconvolution of the CD spectra and reported the rates for the secondary structures’ content in the BSA and their variations in the absence and presence of an excess of U2O in Table 1. 

Far-UV CD spectroscopy provides useful insights into protein conformation analysis, allowing for the monitoring of secondary structure composition and changes after molecular interactions. In fact, the deconvolution of CD spectra into secondary structure compositions allows for the quantification of a given protein α, β-sheet, β-turn and random coil contents and the exploration of the effects of biomolecular interactions on the secondary structure composition based on the resulting CD complex spectra [89,90]. According to Table 1, unliganded BSA showed a 63.0% content of helical structure (comparable to 61% found previously [87,88]) and a 21.7% of β-sheet + β-turn, which was similar to other literature reports [87,88]. On the other hand, U2O provoked significant changes in the secondary structures of BSA, with a certain loss in the helical content (−16.7%), and a concomitant increase in the β-structures (+23.6%) that are indicative of a partial protein destabilization, in agreement with the literature reports [91]. Remarkably, other nitrogen-containing heteroaromatic compounds such as alprazolam were experimentally found to be able to decrease the percentage of BSA α-helical content from 66.5 to 37.0% (Δ_α-helix_ = −29.5%) [92]. Interestingly, the interaction with U2O did not seem to provoke a significant increase in the amount of random coil structure, which indeed resulted in it being slightly decreased (−6.9%, Table 1). Moreover, the ligand-binding could determine modifications in the aromatic regions of BSA, which could explain the slight hyperchromic effect observed in the UV spectrum (Figure 2b), an aspect we investigated in silico as described below. 

### 3.2. Computational Studies 

**Figure 3:**
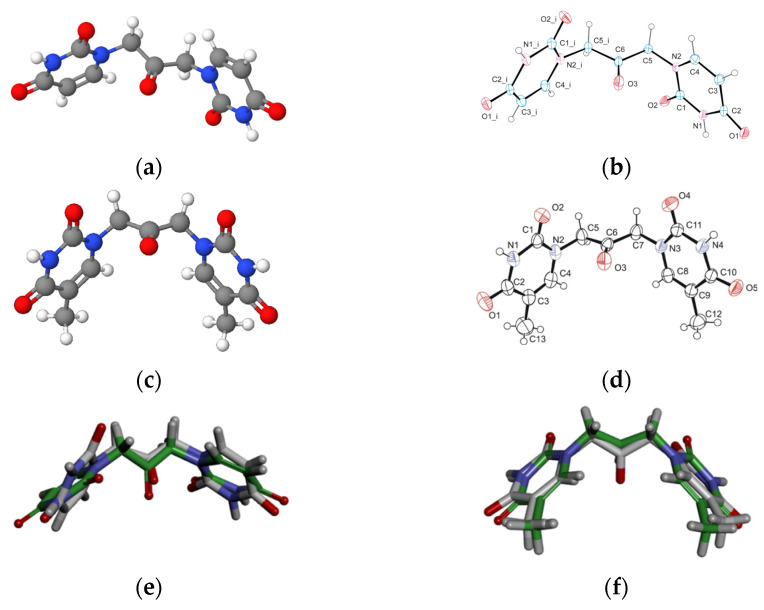
The energy-minimized 3D structure models of U2O (**a**) and T2O (**c**) obtained by MolView software (TU Delft, Deft, Netherlands, v2.4) compared to the X-ray solved structures (**b**,**d**, respectively). Superimposed U2O (**e**) and T2O (**f**) 3D computational models (green) and their X-ray solved structures. The original files for the two X-ray structures can be freely downloaded from https://www.ccdc.cam.ac.uk/ (accessed on 14 July 2022) CCDC 932,679 (U2O) and 821,514 (T2O) [39,41].

**Figure 4:**
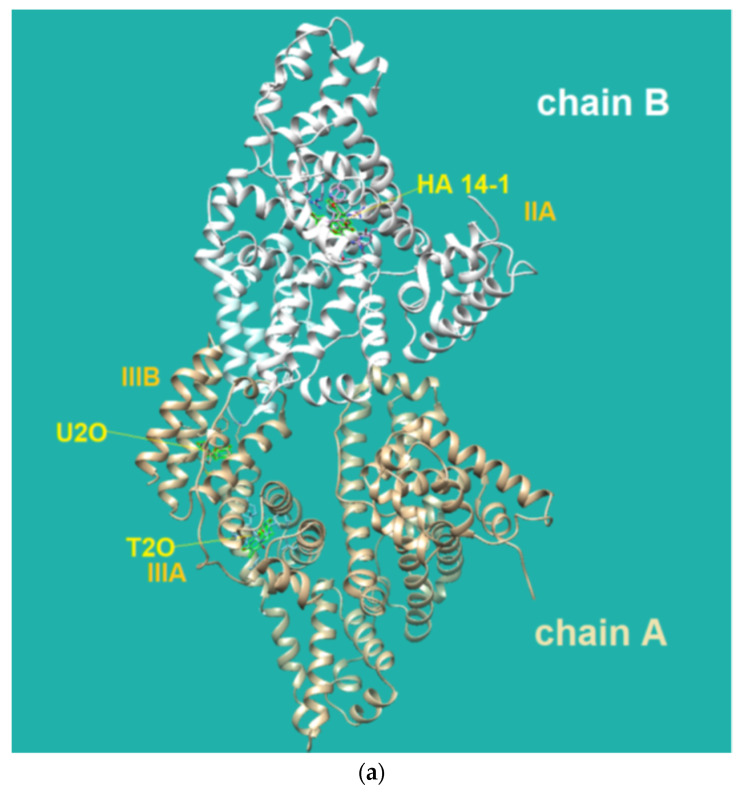

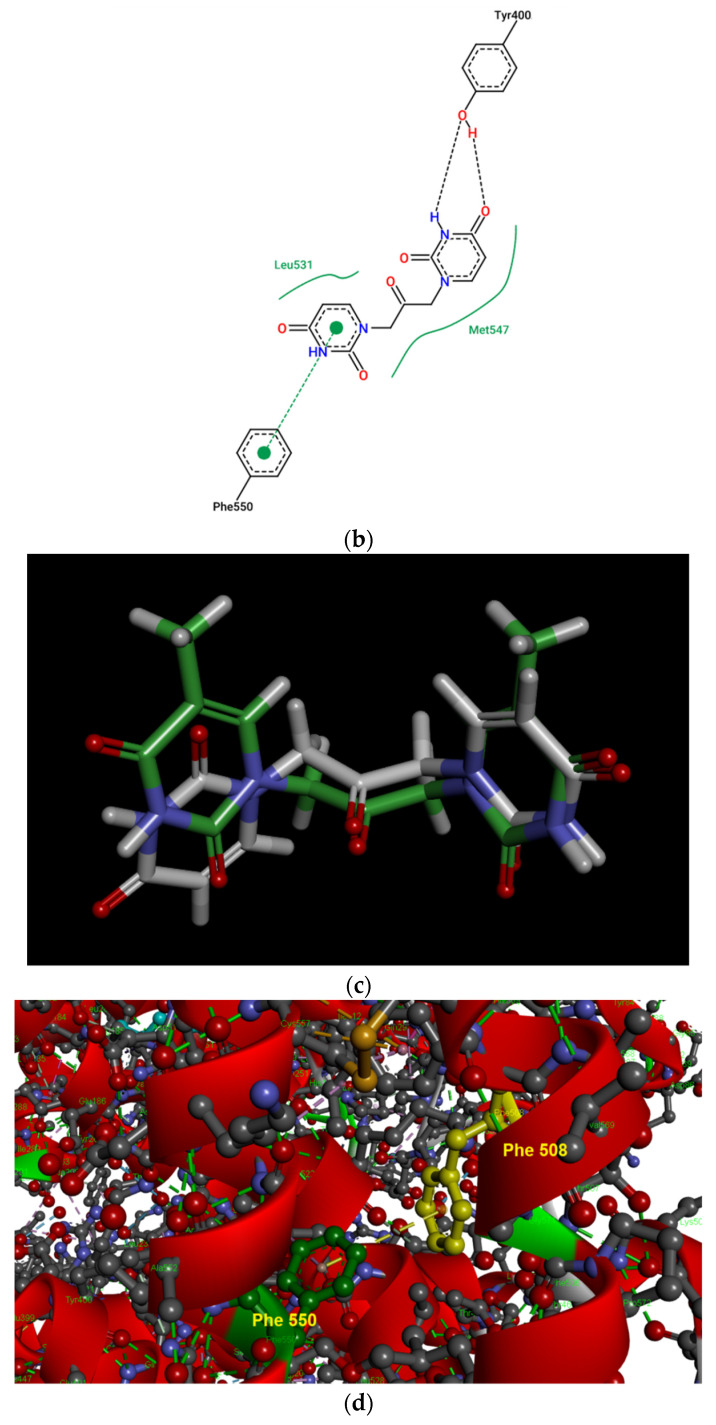
(**a**) Pose view for the complex of U2O with BSA as obtained by blind rigid docking with the HDOCK server and visualized in UCSF Chimera. (**b**) 2D protein–ligand interaction diagram obtained by ProteinPlus with BSA and U2O. H-bond length for C=O—H-O (Tyr 400): 2.44 Å. (**c**) Superimposed 3D models for U2O and T2O. (**d**) Detail of the 3D structure of BSA (PDB ID: 4f5s) as visualized by the software Discovery Studio (Dassault Systèmes Corporate, Waltham, MA, USA, v.2021) showing a π–π stacking interaction between Phe-550 and Phe-508.

Aiming to give a tentative interpretation of some of the binding evidence from our experiments described in the previous sections, we performed a molecular docking study. Within this scope, the HDOCK server was used for blind molecular docking between BSA and U2O. First, we obtained the energy-minimized 3D structure model of U2O by MOLVIEW (http://molview.org, accessed on 30 May 2022), and the 3D structure (Figure 3a) was compared to that (Figure 3b) previously described by some of us through X-ray crystallography that was in good agreement with the predicted structure (Figure 3e) [41]. Similarly, the T2O computational 3D model (Figure 3c) obtained following the same procedure seems to resemble the experimental structure very closely, in terms of the orientation of both heteroaromatic rings as well as C=O, and NH moieties with respect to the central carbonyl moiety (Figure 3d,f). Afterward, the 3D structure of U2O was uploaded as a ligand into the HDOCK server, and the top-10 solutions resulting from the blind rigid docking were analyzed. The results of the docking of U2O with BSA are presented in Table 2 and Figure 4.

The HDOCK scores listed in Table 2 are dimensionless and are helpful only in achieving comparisons between ligands of the same target. Therefore, we also used the same procedure with T2O, which showed a lower affinity for the same protein as revealed by the HDOCK scores and AutoDock Vina binding energies (Table 2). Similarly, we also repeated the blind docking with the reference literature BSA ligand HA 14−1 [73]. Our docking seems to indicate that U2O could bind BSA with higher affinity than HA 14−1, as suggested by the larger negative scores for U2O observed for the top-1, top 1–3, and top 1–10 poses (−158.56 vs. −137.72, −157.11 ± 1.38 vs. −129.80 ± 6.92, −155.18 ± 1.60 vs. −124.72 ± 5.15, respectively, Table 2). Accordingly, the binding energy was more favorable in the case of U2O with respect to T2O and HA 14−1 (−7.6 vs. −7.0 and −5.9 kcal/mol, respectively). Moreover, the amino acid residues involved in the three predicted interactions are different, suggesting that while HA 14−1 binds the chain B of BSA at the subdomain IIA (Figure 4a) as reported in the literature [73], T2O and U2O more likely recognize the albumin (Figure 4a) at the level of chain A, with T2O binding the subdomain IIIA while U2O interacts with Tyr-400 (with predicted H-bonding, C=O—H-O H-bond length: 2.44 Å) and residues such as Phe-550 (π-stacking), Leu-531 and Met-547 (hydrophobic interaction, Figure 4b) of the subdomain IIIB. The differences in BSA binding observed experimentally could be due to the higher steric hindrance determined by the two methyl groups in T2O as well as the different orientation of the carbonyl and NH moieties in this latter compound with respect to U2O (Figure 4c), not allowing the simultaneous H-bond, hydrophobic and aromatic interactions stabilizing the BSA/U2O complex. Our studies suggest that U2O interacts with the protein backbone via hydrogen bonding, and interactions with hydrophobic residues interfere with the mutual attraction of BSA nonpolar groups, as well as π–π stacking interactions leading to the observed UV absorbance differences (e.g., interference with π–π Phe 550–Phe 508 stacking, Figure 4d).

The three-dimensional structure of the serum albumin comprises three helical domains (I, II, and III), each of which is divided into two subdomains (A and B). Several molecules, including warfarin, indomethacin, and phenylbutazone (PB), bind at the binding site IIA, which is also indicated as drug site 1, while others such as diflunisal, diazepam, and iophenoxic acid bind to the site IIIA (also called drug site 2) [73].

**Figure 5:**
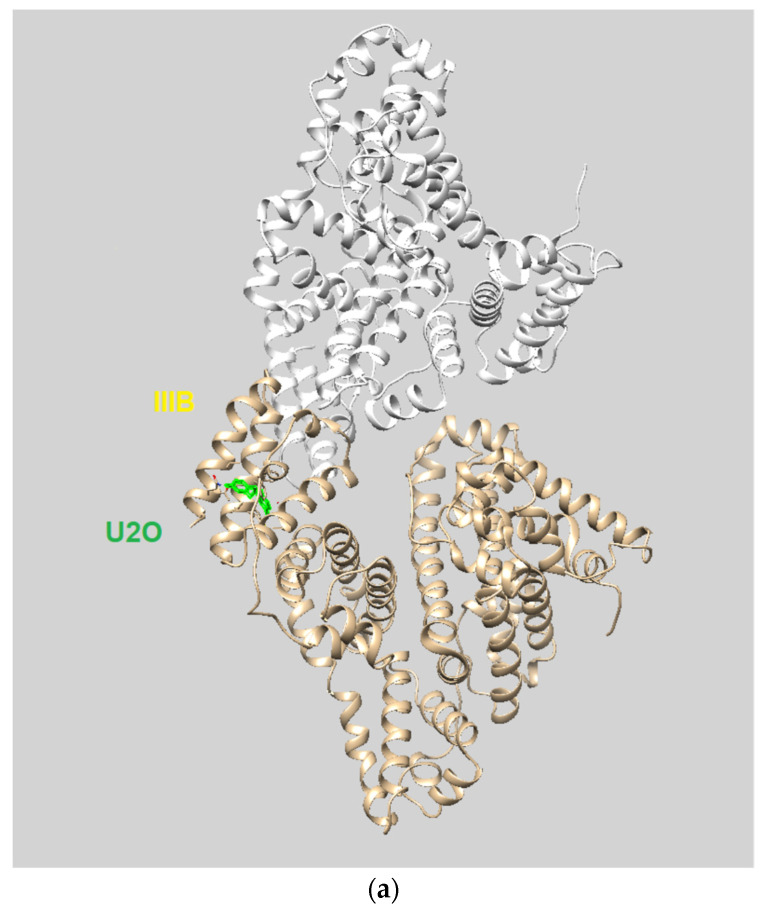

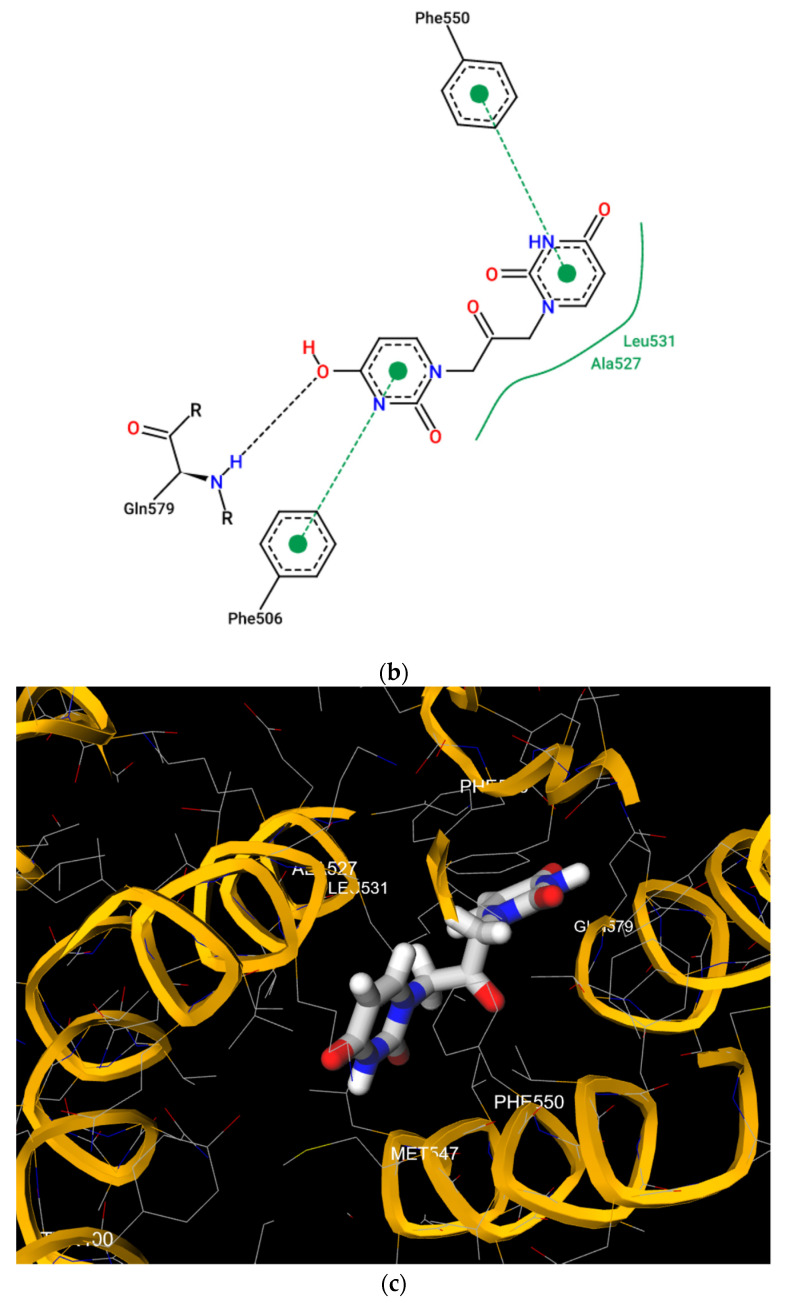
(**a**) Pose view for the complex of U2O with BSA as obtained by blind flexible docking with the FireDock server and visualized in UCSF Chimera. (**b**) 2D protein–ligand interaction diagram obtained by ProteinPlus with BSA/U2O complex after FireDock docking. (**c**) Pose view for the complex BSA/U2O showing the residues involved in the interactions predicted after both the rigid and flexible dockings.

Flexible docking was also performed on U2O in complex with BSA using the program FireDock [79]. This study confirmed the tendency of U2O to bind the albumin in the subdomain IIIB (Figure 5a) involving again Phe-550 in aromatic interactions and Leu-531 (hydrophobic interaction) together with Phe-506 (aromatic interaction), Gln 579 (H-bonding), and Ala-527 (hydrophobic interaction, Figure 5b). Overall, all BSA residues involved in the interaction with U2O predicted after both the rigid and flexible dockings belong to α-helical regions (Figure 5c) of the albumin, suggesting that this molecular recognition can alter the α-helical content of BSA. Finally, in predicting some pharmacokinetics for U2O, we found that the compound, not likely to permeate the blood–brain barrier (BBB, Table 3), is slightly more soluble in water than in organic solvents (cLogP = −0.83, Table 3). Nonetheless, it shows a favorable drug-likeness profile (0 violations of Lipinski’s rule of five, Table 3) [93]. In this prediction, U2O lacks any unspecific biomolecular interaction tendency (PAINS score: 0, Table 3) [94]. On the other side, T2O shows similar predicted pharmacokinetic properties but is endowed with higher hydrophobicity (cLogP = −0.15, Table 3) because of the two methyl moieties of thymine bases.

## 4. Conclusions

We demonstrated that 1,3-bis(1′-uracilyl)-2-propanone (U2O) could bind to serum albumins, which is particularly important in drug delivery applications where these proteins can act as carriers of these bioactive heterocyclic molecules. Our combined experimental (CD- and UV-based) and computational (molecular docking) investigation led us to conclude that the U2O structure can interact with BSA at the level of the subdomain IIIB of the serum albumin in the vicinity of residues such as Phe-550 (π–π stacking), and Leu-531 (hydrophobic interaction) involved in complex formation. In our predictions, the affinity of U2O for BSA was higher than both T2O and the literature ligand HA 14−1 (binding energies: −7.6 vs. −7.0 and −5.9 kcal/mol, respectively). The experimentally found changes in albumin secondary structure content induced by U2O (Δβ-turn + Δβ-sheet = +23.6%, Δα = −16.7%) and the slight hyperchromic effect observed by UV spectroscopy could be explained by the partial protein destabilization and the binding with residues such as Phe-550 that could determine higher exposure of aromatic rings previously involved in π–π stacking with other BSA residues to the solvent, thus increasing the UV absorbance. Overall, all BSA residues involved in the interaction with U2O predicted after both rigid and flexible dockings belong to α-helical regions (Figure 5c) of the albumin, suggesting that this molecular recognition can alter the α-helical content of BSA.

In conclusion, U2O, a building block for the realization of biomedical nanostructures, binds BSA and could likely be efficiently transported in human serum by albumins, thus being effective in the modulation of protein-driven processes. 

## Figures and Tables

**Table 1:** Variation in the BSA secondary structures’ content (%) resulting from the addition of 1,3-bis(1′-uracilyl)-2-propanone. Please note that, besides a ~17% helical content loss detected after ligand binding, U2O determines a significant increase in β-structures.

	Δ(U2O-BSA) (%)	BSA (%)	U2O (%)
**α**	−16.7	63.0	46.3
**β-sheet + β-turn**	+23.6	21.7	45.3
**Random coil**	−6.9	15.3	8.4

**Table 2:** HDOCK docking results for the best pose and mean values from the top-1–3 and top-1–10 poses of U2O, T2O, and reference compound (HA 14−1, [73]) complexed with BSA. The interface residues within 5.0 Å from the ligand in the top-1 complexes are reported in the last column.

	HDOCK Score Top-1 Ranked Pose	HDOCK Score (Top 1–3 Poses) ± SD	HDOCK Score (Top 1–10 Poses) ± SD	Binding Energies (kcal/mol)	Interface Residues
**U2O**	−158.56	−157.11 ± 1.38	−155.18 ± 1.60	−7.6	Tyr 400A, Asn 404A, Leu 505A, Phe 506A, Phe 508A, Lys 524A, Gln 525A, Ala 527A, Leu 528A, Leu 531A, Val 546A, Met 547A, Phe 550A, Val 551A, Leu 574A
**T2O**	−152.59	−149.93 ± 2.35	−142.88 ± 5.61	−7.0	Leu 386A, Ile 387A, Asn 390A, Cys 391A, Phe 394A, Phe 402A, Leu 406A, Arg 409A, Tyr 410A, Lys 413A, Leu 429A, Gly 430A, Lys 431A, Val 432A, Gly 433A, Cys 437A, Thr 448A, Leu 452A, Ser 488A
HA 14−1 *	−137.72	−129.80 ± 6.92	−124.72 ± 5.15	−5.9	Tyr 149B, Glu 152B, Arg 194B, Arg 198B, Trp 213B, Arg 217B, Leu 218B, Lys 221B, Phe 222B, Leu 237B, His 241B, Arg 256B, Ser 286B, Ile 289B, Ala 290B

* reference compound [73].

**Table 3:** Drug-likeness and pharmacokinetic properties predicted for U2O and T2O by the SwissADME software (Swiss Institute of Bioinformatics, Geneva, Switzerland).

**Compound**	**SMILES**	**cLogP**	**BBB** **Perm.**	**Drug-Likeness** (**Lipinski–n. Violations**)	**PAINS**
U2O	O=C(CN1C=CC(=O)NC1=O)CN2C=CC(=O)NC2=O	−0.83	N	Y (0)	N
T2O	Cc2cn(CC(=O)Cn1cc(C)c(=O)[nH]c1=O)c(=O)[nH]c2=O	−0.15	N	Y (0)	N
